# Toxic metabolites, Sertoli cells and Y chromosome related genes are potentially linked to the reproductive toxicity induced by mequindox

**DOI:** 10.18632/oncotarget.20916

**Published:** 2017-09-15

**Authors:** Qianying Liu, Zhixin Lei, Menghong Dai, Xu Wang, Zonghui Yuan

**Affiliations:** ^1^ National Reference Laboratory of Veterinary Drug Residues (HZAU) and MAO Key Laboratory for Detection of Veterinary Drug Residues, Huazhong Agricultural University, Wuhan, Hubei, China; ^2^ MOA Laboratory for Risk Assessment of Quality and Safety of Livestock and Poultry Products, Huazhong Agricultural University, Wuhan, Hubei, China; ^3^ Hubei Collaborative Innovation Center for Animal Nutrition and Feed Safety, Wuhan, Hubei, China

**Keywords:** mequindox, reproductive toxicity, blood-testis barrier, Sertoli cells, Y chromosome microdeletion

## Abstract

Mequindox (MEQ) is a relatively new synthetic antibacterial agent widely applied in China since the 1980s. However, its reproductive toxicity has not been adequately performed. In the present study, four groups of male Kunming mice (10 mice/group) were fed diets containing MEQ (0, 25, 55 and 110 mg/kg in the diet) for up to 18 months. The results show that M4 could pass through the blood-testis barrier (BTB), and demonstrate that Sertoli cells (SCs) are the main toxic target for MEQ to induce spermatogenesis deficiency. Furthermore, adrenal toxicity, adverse effects on the hypothalamic-pituitary-testicular axis (HPTA) and Leydig cells, as well as the expression of genes related to steroid biosynthesis and cholesterol transport, were responsible for the alterations in sex hormones in the serum of male mice after exposure to MEQ. Additionally, the changed levels of Y chromosome microdeletion related genes, such as DDX3Y, HSF2, Sly and Ssty2 in the testis might be a mechanism for the inhibition of spermatogenesis induced by MEQ. The present study illustrates for the first time the toxic metabolites of MEQ in testis of mice, and suggests that SCs, sex hormones and Y chromosome microdeletion genes are involved in reproductive toxicity mediated by MEQ *in vivo*.

## INTRODUCTION

**Figure 1:**
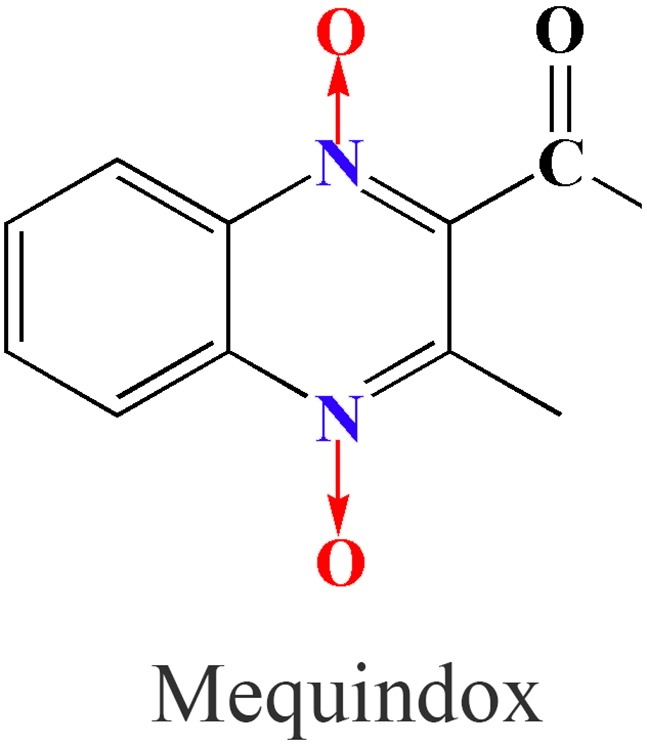
Chemical structure of mequindox (MEQ)

Mequindox (3-methyl-2-acetyl-N-1,4-dioxyquinoxaline, C_11_H_10_N_2_O_3_; MEQ) (Figure 1), structurally similar to other classical members of the quinoxaline-di-*N*-oxides (QdNOs) [1], is a relatively new synthetic antibacterial agent that was developed by the Lanzhou Institute of Animal Husbandry and Veterinary Drugs at the Chinese Academy of Agricultural Sciences [2]. MEQ was shown to be better than other antimicrobial agents for the treatment of swine dysentery (*T. hyodysenteriae*) at a dose of 5-10 mg/kg b.w., and it has been widely applied in pigs and chickens in China owing to its efficacious in treatment of clinical infections caused by *Treponeme, Pasteurella, Staphylococcus aureus, E. coli*, and *Salmonella* sp. [3–6].

Serious health hazard effects have been noted when QdNOs was used as an animal feed additive or antimicrobial agent [2]. Considerable evidence suggests that carbadox (CBX) and olaquindox (OLA) are mutagenic and carcinogenic with developmental and reproductive toxicities [5, 7–10]. Previous studies revealed that teratogenic effects and significant decreases in fetal and maternal body weights were observed in rats after exposure to CBX at doses as low as 25 mg/kg per day [9]. OLA was reported to be responsible for toxic effects in the testes of rats after administration at a dose of 5 mg/kg b.w./day [8]. MEQ and its primary metabolites, *N*1-desoxymequindox (*N*1-MEQ) and bidesoxy-mequindox (B-MEQ), exhibited adrenal toxicity in H295R cells that originated from a human adrenocortical carcinoma [11], and showed genotoxicity in short-term *in vitro* and *in vivo* tests [5, 12]. The *in vivo* toxicity study of MEQ in rodents demonstrated that it resulted in obvious histological changes in the adrenal gland [13], liver [1, 14] and spleen [14]. In the reproductive system, long-term MEQ treatment induced endocrine and reproductive toxicity via oxidative stress in male Wistar rats [4]. MEQ was reported to disrupt spermatogenesis with a decreased number of sperm and an increased number of morphologically abnormal sperm [15]. However, the underlying mechanisms of the reproductive toxicity of MEQ *in vivo* still remain unclear.

Traditionally, the function of the blood-testis barrier (BTB), one of the tightest blood-tissue barriers, is to facilitate spermiogenesis and spermiation by preventing the diffusion of various endogenous and exogenous toxic substances from entering apical compartments during the epithelial cycle [16–20]. Dysfunction of the BTB will lead to interference in spermatogenesis and infertility [21, 22]. Tight junctions (TJs) that consist of Sertoli cells (SCs) are one of the most important components in the formation of the BTB and provide a microenvironment for spermatogonial stem cells [23, 24]. Many studies have shown the critical role of SCs in the permeability of the BTB [25] and spermatogenesis [25–27]. Regarding the reproductive toxicity induced by MEQ, we recently found that MEQ causes adverse effects on spermatogenesis and the integrity of the BTB with tight junctions (TJs) as the macromolecular target. Therefore, it is suspected that SCs may be involved in the disruption of the BTB and spermatogenesis deficiency caused by MEQ *in vivo*. A few studies have revealed that the Y chromosome carries a large number of reproduction-related genes that are responsible for spermatogenesis; the deletion of Y chromosome microdeletion genes directly results in the failure of spermatogenesis [28–33]. Thus, apart from the SCs, the role of Y chromosome microdeletion genes in spermatogenesis mediated by MEQ *in vivo* should also be investigated.

Based on the above information, the objective of this study was to clarify the reproductive toxicity of MEQ *in vivo*, and to further elucidate the role of SCs and Y chromosome microdeletion genes in reproductive toxicity induced by MEQ *in vivo*. In the present study, we comprehensively evaluated reproductive toxicity in Kunming mice after exposure to MEQ for 18 months. To verify the above hypothesis, we investigated the histology and ultrastructure of testis, metabolites of MEQ in the serum and testis, serum sex hormone concentration as well as the mRNA expression levels of Y chromosome microdeletion related genes and the genes responsible for steroid biosynthesis and cholesterol transport in the testes of mice. This study is of great significance to evaluate MEQ in its clinical use and improve the prudent use of QdNOs for public health.

## RESULTS

### Body weight, testes coefficient and MEQ intake

**Figure 2:**
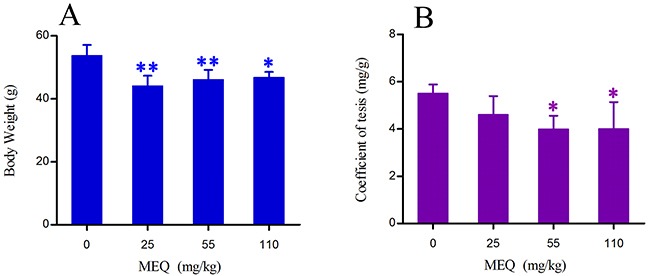
Body weight **(A)** and coefficient of testis **(B)** in male mice after the administration of MEQ for 18 months. ^*^ *p*< 0.05, and ^**^p< 0.01 in comparison with control. Values represent mean ± SD (n = 10).

The final body weight and testes coefficient of male mice after the administration of MEQ for 18 months are shown in Figure 2. The coefficient of testes to body weight was expressed as milligrams (wet weight of testes mg)/(grams body weight, g). Compared with the control group, there was a significant decrease in body weight in 25, 55 (*p*< 0.01), and 110 mg/kg (*p*< 0.05) groups. A significant reduction in the coefficient of testes was noted in the 55 and 110mg/kg groups (*p*< 0.05) as compared with controls.

MEQ intakes per kg body weight per day (mg/kg b.w./day) were calculated based on feed intake and body weight. The results are shown in Table 1.

**Table 1:** Daily intake in study of mequindox in male Kun ming mice (mean±SD)

Group number^a^	Treatment	Concentrations (mg/kg diet)	Doseage^b^ (mg/kg b.w./day)
1	Basal diet	Control	0
2	Mequindox	M25	1.71 ± 0.12
3	Mequindox	M55	3.58 ± 0.24
4	Mequindox	M110	7.07 ± 0.26

*Note*: SD, standard deviation; ^a^10 mice/group; ^b^counted from weekly individual feed consumptions and nominal dietary concentration; M, mequindox; M25, 25 mg/kg diet; M55, 55 mg/kg diet; M110, 110 mg/kg diet.

### Histological evaluation

**Figure 3:**
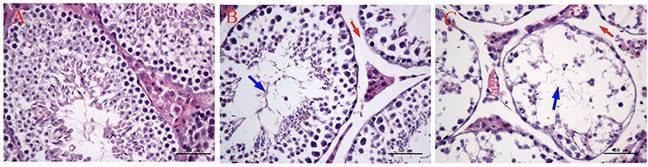
Selected microphotographs of testes in male mice after the administration of MEQ for 18 months (400×) **(A)** Testes from the control group. **(B)** Testes from the 55 mg/kg MEQ group. Red arrow shows broadened interstitial testicular tissue and blue arrow shows decreased number of spermatogenic cells in the lumen. **(C)** Testes from the 110 mg/kg MEQ group. Red arrow shows broadened interstitial testicular tissue and blue arrow shows necrosis of spermatogonia and spermatocytes in the lumen.

As shown in Figure 3, there were significant histopathological changes in testes after exposure to MEQ. In the control testes, a normal testicular interstitium and a greater number of developing sperm cells were observed in seminiferous tubules (Figure 3A). Compared with the control group, a broadened testicular interstitium and an irregular arrangement as well as a decreased number of spermatogenic cells in the lumen were noted in the 55mg/kg group (Figure 3B). In the 110mg/kg group, spermatogonia and spermatocytes in the lumen exhibited necrosis and disorganization of the germinal epithelium (Figure 3C).

### Ultrastructural alterations in the testis

**Figure 4:**
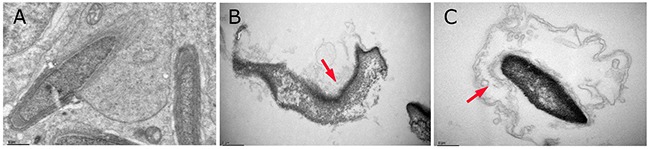
Ultrastructure of sperm in male mice after the administration of MEQ for 18 months (scale bar = 1 μm) **(A)** Sperm from the control group. **(B)** Sperm from the 55 mg/kg MEQ group. Arrow shows abnormal morphology, fracture and dissolution. **(C)** Sperm from the 110 mg/kg MEQ group. Arrow shows abnormal morphology and dissolution of the membrane.

**Figure 5:**
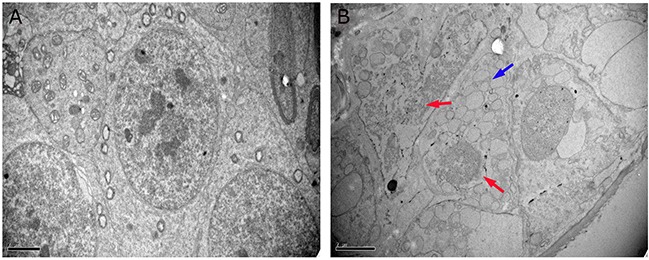
Ultrastructure of SCs in male mice after the administration of MEQ for 18 months (scale bar = 2 μm) **(A)** SCs from the control group. **(B)** SCs from the 110 mg/kg MEQ group. Blue arrow shows a few vacuoles in cytoplasm and red arrows show the dissolution and fragmentation of nuclear.

The ultrastructural changes in sperm (Figure 4) and SCs (Figure 5) in the testes of male mice after the administration of 25, 55 and 110 mg/kg MEQ for 18 months were analyzed by transmission electron microscopy (TEM). In the control group, the sperm in mice testes showed an integral membrane structure and normal size (Figure 4A); the SCs contained a round nucleus with homogeneous chromatin (Figure 5A). In the MEQ treated groups, the sperm appeared to be morphologically abnormal, including enlarged with breaking and dissolving locally in the 55mg/kg group (Figure 4B), and with cell membrane lysis in the 110mg/kg group (Figure 4C). The ultrastructure of SCs from the 110 mg/kg diet MEQ group indicated necrosis with dissolved cell membranes and nuclear fragmentation (Figure 5B). These results indicate the potential interference of MEQ in spermatogenesis and the integrity of the TJs.

### MEQ and its metabolites in serum and testes

**Figure 6:**
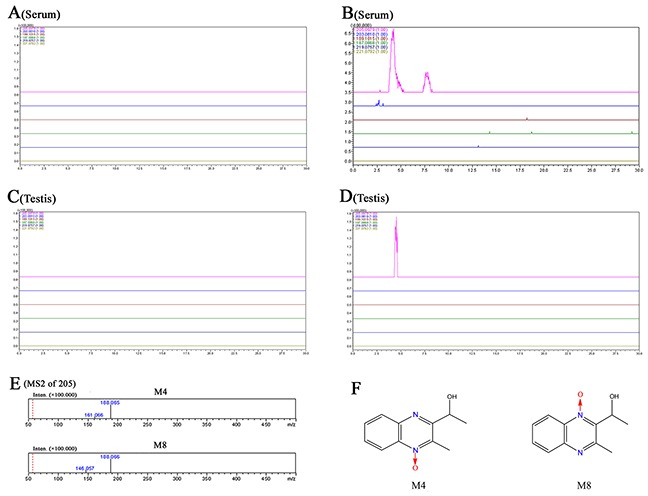
Accurate EICs of the prototype and metabolites of MEQ in the serum and testes of male mice by LC/MS-IT-TOF analysis **(A)** Serum from the control group. **(B)** Serum from the 110 mg/kg MEQ group. **(C)** Testis from the control group. **(D)** Testis from the 110mg/kg MEQ group. **(E)** The accurate MS2 spectra of M4 and M8, respectively. **(F)** The chemical structure of M4 is 2-isoethanol 1-desoxymequindox, and M8 is 2-isoethanol 4-desoxymequindox.

The prototype and metabolites of MEQ in serum and testes were detected by LC/MS-IT-TOF analysis according to the retention times and fragment ions. M4 and M8 were confirmed as 2-isoethanol 1-desoxymequindox and 2-isoethanol 4-desoxymequindox, respectively, according to the accurate MS^2^ spectra (Figure 6E). The results show that no MEQ prototype was found in the serum and testis. Two metabolites (M4 and M8) were detected in the serum in the 110 mg/kg MEQ group (Figure 6B), while only one metabolite (M4) was found in the testis after the administration of MEQ at a dose of 110 mg/kg for 18 months (Figure 6D). These results suggest that M4 may be a biomarker of reproductive toxicity induced by MEQ in mice, which induces sperm aberration via disrupt the function of SCs as evidenced by our observations in TEM analysis.

### Serum sex hormone

**Figure 7:**
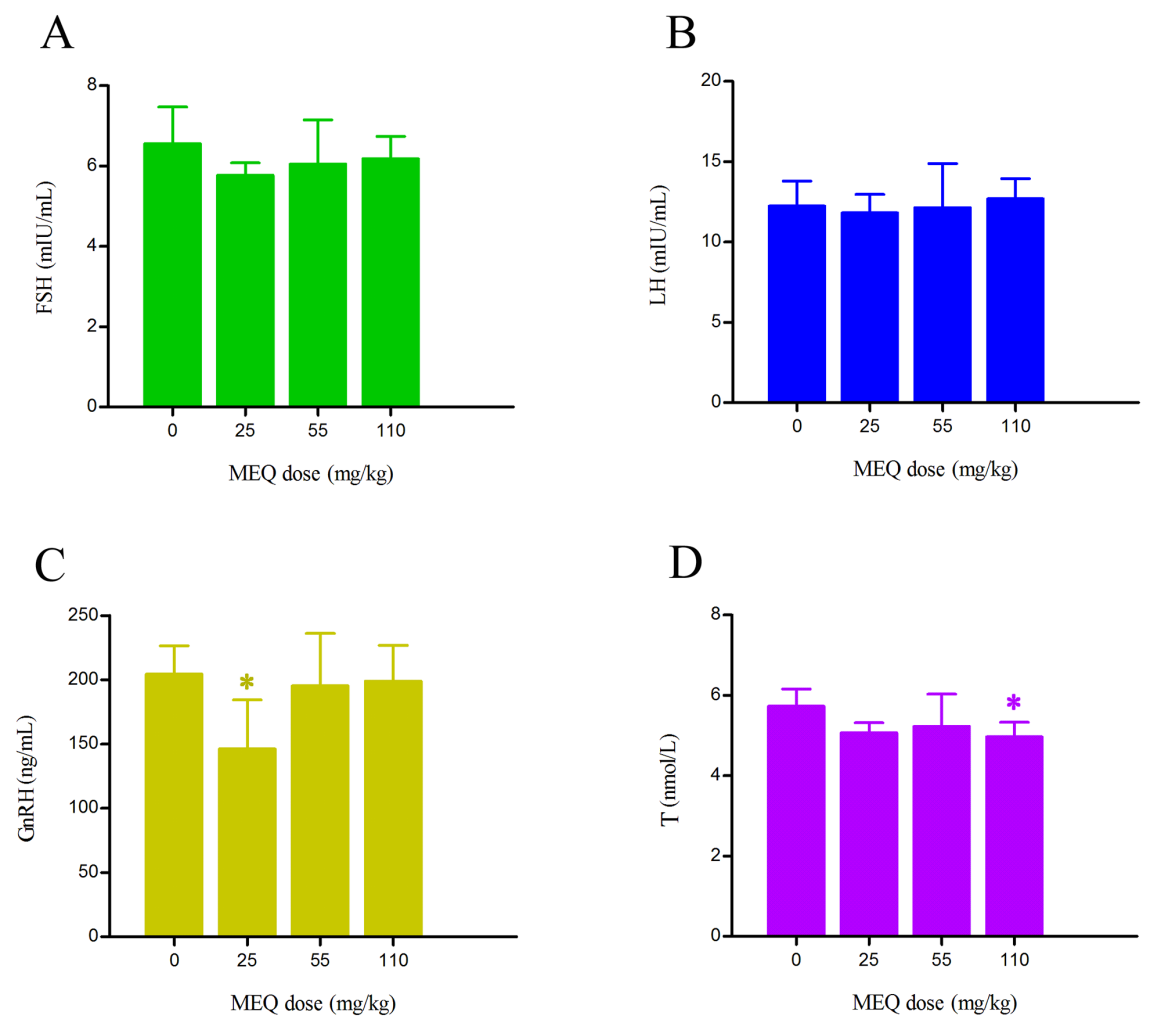
Alterations to hormonal levels of **(A)** FSH (mIU/mL), **(B)** LH (mIU/mL), **(C)** GnRH (ng/mL), and **(D)** T (nmol/L) in the serum of male mice after the administration of MEQ for 18 months. ^*^ *p*< 0.05, and ^**^ *p*< 0.01 in comparison with control. Values represent mean ± SD (n = 10).

In this study, four sex hormones in serum, including follicle-stimulating hormone (FSH), luteinizing hormone (LH), testosterone (T), and gonadotropin-releasing hormone (GnRH) were measured and shown in Figure 7. A downward tendency of FSH, GnRH, and T in the MEQ exposure groups was noted as compared to the control group. Significantly decreased levels of GnRH and T were observed in the 25 and 110 mg/kg group (*p*< 0.05), respectively. There was no significant differences in the level of FSH in MEQ treated groups (*p*> 0.05). Compared with the control group, the level of LH was higher in the 110mg/kg group, and lower in the 25 and 55 mg/kg groups, but the differences were not significant (*p*> 0.05). These findings demonstrate that change in the levels of sex hormones may be associated with the effect of MEQ on the hypothalamic-pituitary-testicular axis (HPTA).

### The mRNA expression of T hormone synthesis and spermatogenesis related genes

**Figure 8:**
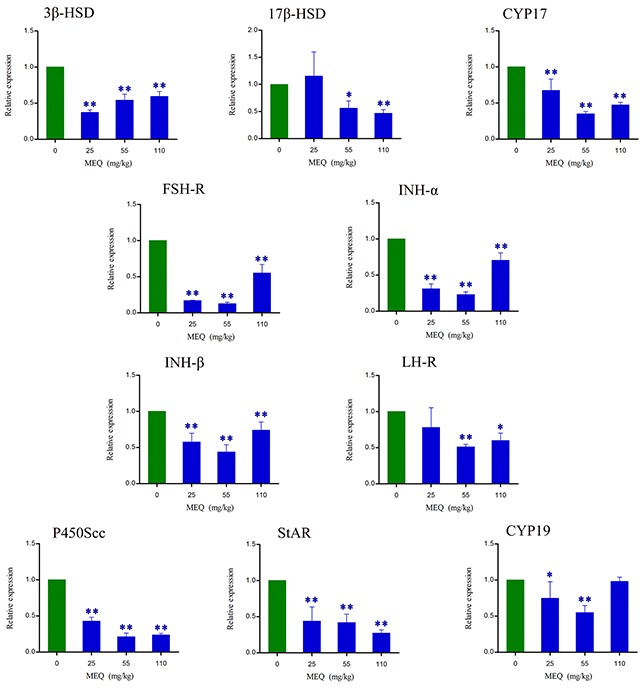
Alterations in 3β-HSD, 17β-HSD, CYP17, FSH-R, INH-α, INH-β B, LH-R, P450Scc, StAR, and CYP19 expression in mouse testes after the administration of MEQ for 18 months ^*^ *p*< 0.05, and ^**^ *p*< 0.01 in comparison with control. Values represent mean ± SD (n = 10).

The effects of MEQ on the mRNA expression of genes related to spermatogenesis (e.g. FSH-R, INH-α, and INH-β B), and cholesterol transport and steroidogenesis (e.g. 3β-HSD, 17β-HSD, CYP17, LH-R, P450Scc, StAR, and CYP19) in male mice were determined using real-time quantitative RT-PCR. Figure 8 shows that exposure to MEQ induced significant decreases in 3β-HSD, CYP17, FSH-R, INH-α, INH-β B, P450Scc, and StAR expression in the testes (*p*< 0.01). With increased MEQ doses, there was a significant increase in 3β-HSD expression (*p*< 0.01) and a significant decrease in 17β-HSD (*p*< 0.05 or *p*< 0.01) and StAR expression (*p*< 0.01). Decreased mRNA levels of 17β-HSD and LH-R were observed at the doses of 55 and 110 mg/kg (*p*< 0.05 or *p*< 0.01), and CYP19 at the doses of 25 and 55 mg/kg (*p*< 0.05 or *p*< 0.01).

### The mRNA expression of Y chromosome related genes

**Figure 9:**
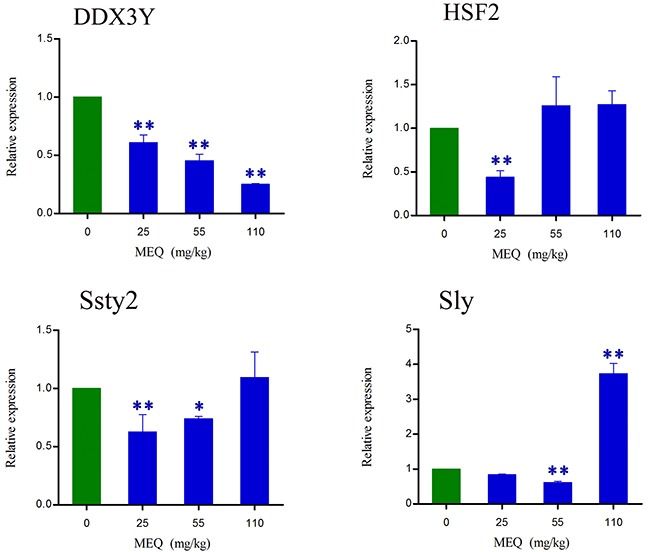
lterations in DDX3Y, HSF2, Ssty2, and Sly expression in mouse testis after the administration of MEQ for 18 months ^*^ *p*< 0.05, and ^**^ *p*< 0.01 in comparison with control. Values represent mean ± SD (n = 10).

To investigate whether prolonged treatment with MEQ in mice affects spermatogenesis and male fertility by alterations in the expression of Y chromosome microdeletions genes, some related genes (e.g. DDX3Y, HSF2, Ssty2, and Sly) in male mice were assessed using real-time quantitative RT-PCR (Figure 9). As shown in Figure 9, a significant decrease in DDX3Y expression (*p*< 0.01) in male mice was noted after the administration of MEQ for 18 months. Decreased mRNA expression of HSF2 and Sly were observed in the 25 and 55 mg/kg group (*p*< 0.01), respectively. A significant increase in the expression of Sly was noted in the 110 mg/kg group (*p*< 0.01). There was markedly reduced expression of Ssty2 in the 25 and 55 mg/kg groups (*p*< 0.05 or *p*< 0.01). These results indicate that the changed expression of Y chromosome related genes may responsible for the adverse effect on spermatogenesis and sperm development mediated by MEQ in mice.

## DISCUSSION

Due to the significant antibacterial properties and growth promotion ability, MEQ was widely developed for use in animal productions in China since the 1980s [2, 36–38]. However, its toxicological evaluation, especially regarding reproductive toxicity, has not been adequately performed. In the present study, we comprehensively evaluate the reproductive toxicity in Kunming mouse after exposure to MEQ for 18 months. The results demonstrate that the metabolites of MEQ, sex hormones, the SCs, and Y chromosome related genes are all involved in reproductive toxicity in male mice after chronic administration of MEQ. Additionally, M4 was found to pass through the BTB. Adrenal toxicity, adverse effects on the HPTA and Leydig cells, and changes in the mRNA expression of genes related to steroid biosynthesis and cholesterol transport, were responsible for the alterations of sex hormones in the serum of mice after exposure to MEQ. Furthermore, Y chromosome related genes, such as DDX3Y, HSF2, Sly, and Ssty2 in the testis, might be involved in the inhibition of spermatogenesis induced by MEQ. Importantly, the present study demonstrates that SCs might be one of the key targets by which MEQ induces a disruptive effect on TJs and spermatogenesis.

Previous studies have documented that MEQ exposure induces reproductive toxicity [4]. MEQ was found to alter the growth and development of the next generation with decreased fertility and changes in sperm morphology [15]. In the present study, reductions in body weight and the coefficient of the testes were observed when male mice were exposed to MEQ (25, 55, 110 mg/kg) for 18 months (Figure 2). The histopathological evaluation of MEQ treated mice showed obvious damage to the testes, such as a broadened testicular interstitium, disorganization of the germinal epithelium, and necrosis of both spermatogonia and spermatocytes in the lumen (Figure 3). In the TEM analysis, the sperm exhibited dissolved cell membranes and enlarged with fracture in the MEQ treated groups (Figure 4). It has been revealed that the BTB is associated with male fertility by physically dividing the seminferous epithelium into basal and apical compartments [16–18]. The function of SCs, one of the primary somatic cells in the seminiferous epithelium, is to provide a microenvironment for spermatogonial stem cells through the secretion of specific factors [26]. SCs play a critical role in the permeability of the BTB [25] and spermatogenesis [25–27]. Here, the ultrastructural observation of SCs in the 110 mg/kg MEQ group showed nuclear fragmentation and dissolved cell membranes in the TEM analysis (Figure 5), suggesting impaired integrity of TJs induced by MEQ. These findings not only further confirm earlier findings that testis damage and increased sperm malformation occurred after chronic oral administration of MEQ [4], but also reveals that SCs might be a main target for MEQ to induce TJ disruption and subsequently, cause the toxicity to spermatogenesis, sperm development and sperm maturation.

The *N*→O group reduction metabolites of MEQ were found to be associated with its toxicity. The primary metabolites of MEQ–*N*1-MEQ and B-MEQ, exhibit genotoxicity in short-term *in vitro* and *in vivo* tests [12]. The metabolites of MEQ also induce toxicity *in vivo* in the adrenal glands [13], liver and spleen [14], and reproductive system [4]. M11 was identified as the main toxic metabolite in the liver and spleen of Wistar rats after exposure to MEQ for 180 days [14]. Recently, M4 and M8 were recognized as toxic metabolites in the liver of mice after the administration of MEQ for 11 months [1]. As many studies suggested, QdNOs derivatives were used as growth promoters in husbandry because of their strong antimicrobial activity [1, 2, 4, 5, 12]. However, up to now, the antimicrobial activity of QdNOs has mainly focused on the parent drugs and subsequent reactive oxygen species (ROS) and hydroxyl radicals from the bacterial metabolism of QdNOs [63]. It still remains undiscovered whether their metabolites have antibacterial activity and further study should be conducted to focus on this issue.

In this study, two metabolites of MEQ (M4 and M8) were found in the serum of mice by LC/MS-IT-TOF analysis (Figure 6B), while only M4 appeared in the testis (Figure 6D), indicating that M4 can pass through the BTB to disrupt the reproductive system directly. This finding explains the ultrastructural observations in SCs and sperm, suggesting that transport M4 from the serum to the testis might be an important factor in the toxic effects on TJs integrity, spermatogenesis and sperm development. Additionally, M4 detected in the testis of male mice demonstrates that M4 may act as a biomarker of reproductive toxicity induced by MEQ. These results provide new insight into the firm connection of metabolism of a drug with its organ toxicity. Therefore, further studies should focus on the role of M4 on spermatogonia and spermatocytes.

**Figure 10:**
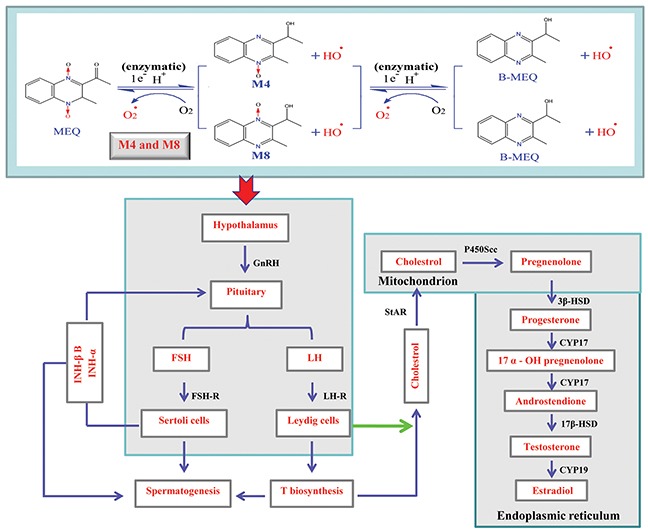
Hypothalamic-pituitary-testicular axis (HPTA), testosterone (T) biosynthetic pathway and potential metabolism of MEQ in the male mice The schematic diagram indicates the structure and functions of HPTA (spermatogenesis and T biosynthesis), and shows the reactions leading from cholesterol to T including the originating cell (Leydig cells), corresponding enzymes, and organelles in which the reactions are carried out. 17β-HSD, 17-beta-hydroxysteroid dehydrogenase; 3β-HSD, 3-beta-hydroxysteroid dehydrogenase; CYP17, cytochrome P450c17 subfamily a; CYP19, cytochrome P450c19 subfamily a; FSH, follicle-stimulating hormone; LH, luteinizing hormone; FSH-R, follicle-stimulating hormone receptor; GnRH, gonadotropin-releasing hormone (GnRH); INH-α, inhibin subfamily α; INH-β B, inhibin subfamily β B; LH-R, luteinizing hormone receptor; P450scc, cholesterol side-chain cleavage enzyme; StAR, steroidogenic acute regulatory protein.

**Figure 11:**
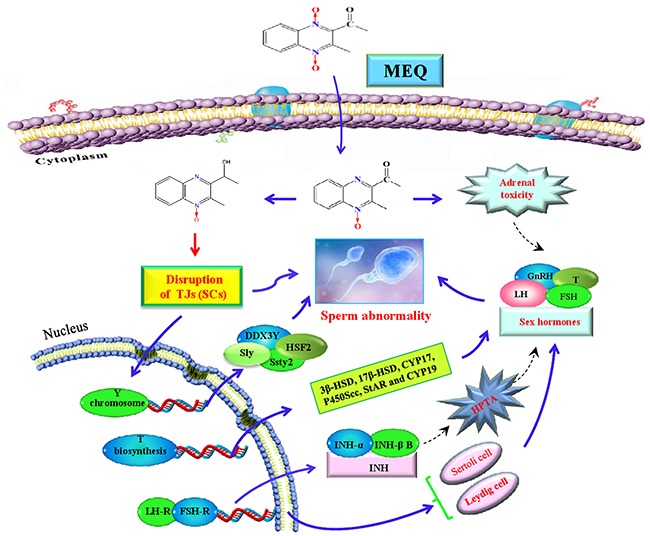
The proposed mechanisms of reproductive toxicity in mouse caused by MEQ SCs are the main toxic target for MEQ to induce TJ disruption and morphologically abnormal sperm. The disrupted TJs further result in the transport of M4 into the testis where it exerts toxic effects on spermatogenesis. Additionally, Y chromosome microdeletion related genes, such as DDX3Y, HSF2, Sly, and Ssty2 in the testis play important roles in the MEQ-induced inhibition of spermatogenesis. Moreover, the MEQ-mediated alteration of sex hormones may result from adrenal toxicity, adverse effects on the HPTA, Leydig cells and SCs, as well as the down-regulated expression of genes responsible for steroid biosynthesis and cholesterol transport. Briefly, SCs, Y chromosome microdeletion related genes and changes to sex hormones levels are involved in the reproductive toxicity mediated by MEQ *in vivo*.

Regarding serum sex hormones, the function of FSH and LH is to promote testicular steroidogenesis and secrete T [39–43]. T is required in the normal morphology of seminiferous tubules and spermatogenesis [40–41, 44]. GnRH, one of the most important neuropeptides released by hypothalamus, stimulates FSH and LH pulsatile release from the pituitary [40, 45] (Figure 10). All of these hormones are controlled by the hypothalamus-pituitary-testicular axis (HPTA) through a negative feedback mechanism [40, 46]. In the current experiment, our data show a tendency toward changes in the levels of FSH and LH, a decreased level of GnRH in 25mg/kg group, and decreased T in the 110mg/kg group (Figure 7). We suspect that the changes in serum sex hormones may be a secondary phenomenon of the interference in the HPTA mediated by MEQ. The connection between sperm abnormalities and sex hormones is well-recognized [47–49]. Herein, the changes to sex hormones support the defects in sperm morphology induced by MEQ. Commonly, alterations in sex hormone and the steroidogenic pathway are associated with the function of adrenal cells [50]. Previous studies have revealed that MEQ caused adrenal toxicity *in vitro* [11, 51] and *in vivo* [13, 52]. Additionally, the metabolites of MEQ, such as M4 and M8, were detected in the serum of male mice (Figure 6B). Therefore, apart from the HPTA, adrenal toxicity and MEQ metabolites (M4 and M8) may be another reason for the changes in sex hormone induced by MEQ (Figure 10 and Figure 11).

It is believed that the cooperation of some functional molecules, including 3β-HSD, 17β-HSD, CYP17, P450Scc, StAR and CYP19, are involved in T biosynthesis to complete the process of steroid biosynthesis and cholesterol transport [4, 41, 53]) (Figure 10). Steroid biosynthesis is stimulated when LH binds to LH-R on the surface of Leydig cells [40, 54, 55]. The mRNA expression of LH-R was significantly decreased in the MEQ treated groups (Figure 8). Additionally, the level of LH in the serum was reduced in the 25 and 55 mg/kg groups (Figure 7). These results demonstrate that MEQ induces changes to steroid biosynthesis, and the dysfunction of Leydig cells may be a critical step to trigger this toxicity. The function of StAR is to transport cholesterol from the outer mitochondrial membrane to the inner mitochondrial membrane in Leydig cells; cholesterol is then converted into pregnenolone by P450scc [41] (Figure 10). Pregnenolone in the endoplasmic reticulum is converted into T by 3β-HSD, CYP17 and 17β-HSD (Figure 10). CYP19 is required to induce the conversion of T into estradiol [41]. In this study, we found a decreased tendency in the mRNA expression of StAR, P450Scc, 3β-HSD, CYP17, 17β-HSD, and CYP19 (Figure 8), suggesting that cholesterol transport and steroidogenesis are adversely affected by MEQ. Altogether, the pathways responsible for steroid biosynthesis and cholesterol transport, and the dysfunction of Leydig cells were involved in decreased secretion of T in male mice after chronic exposure to MEQ.

The maintenance of a high concentration of T requires an androgen binding protein that is synthesized when SCs are activated by FSH [4]. Therefore, the decreased secretion of FSH and T in this study may result from its adverse effect on SCs. This result is consistent with the ultrastructural changes of SCs observed in the TEM analysis (Figure 5). Inhibin B, a member of the inhibins family, is a main factor regulating the secretion of FSH in the testis [40, 56] (Figure 10). The signaling pathway responsible for spermatogenesis is activated when FSH binds to the FSH-R on the surface of SCs [40, 54, 55] (Figure 10). Here, the down-regulated mRNA expression of INH-α, INH-β B, and FSH-R indicates a possible mechanism of the inhibition of FSH. Additionally, the decreased level of FSH and the reduced mRNA expression of FSH-R demonstrate the inhibition of spermatogenesis. Moreover, the present data further reveal that SCs are an important toxicity target for MEQ-induced adverse effects on spermatogenesis and the secretion of sex hormones. Therefore, the reproductive toxicity of MEQ is firmly associated with alterations in the function and structure of SCs in the testis. Further studies should be conducted to investigate the role of SCs in reproductive toxicity after exposure to MEQ.

Spermatogenesis requires the expression of sperm-specific genes. As is already known, during spermatogenesis, the paternal DNA of the oocyte is provided by differentiated pluripotent germ cells in a steady stream. Previous studies indicated that the deletion of the male-specific region of the mouse Y chromosome resulted in the failure of spermatogenesis [28, 29, 33]. DDX3Y, located in the AZFa interval in the Y chromosome, has been found to play an important role in regulating spermatogenesis and sperm development [28, 32]. In the present study, a significant decrease in DDX3Y expression in male mice was noted in MEQ-treated groups, demonstrating that DDX3Y is an important target gene for the reproductive toxicity mediated by MEQ. Sly and Ssty2, located in the male specific region of MSYq, are specifically expressed during spermatogenesis [57], while HSF2 is mainly expressed in differentiated male germ cells [58]. It has been reported that the target genes of HSF2 include Ssty2 and Sly [28, 58, 59]. In this study, reduced mRNA expression of HSF2 was noted in the 25 mg/kg group, consistent with the decreased mRNA expression of Ssty2 and Sly in this group. A deficiency in Ssty2, Sly, and HSF2 was related to an increase in the sperm head abnormality ratio [58, 60–62]. Collectively, down-regulated DDX3Y, HSF2, Sly, and Ssty2 in the testis might be the mechanism responsible for the MEQ-induced inhibition of spermatogenesis and ultrastructural changes in sperm.

In conclusion, the present study demonstrates that M4 is a potential biomarker of reproductive toxicity induced by MEQ in mice. SCs are the main toxic target for MEQ to induce TJs disruption and development of morphologically abnormal sperm. Additionally, Y chromosome microdeletion related genes, such as DDX3Y, HSF2, Sly, and Ssty2 in the testis might be the mechanism responsible for the inhibition of spermatogenesis caused by MEQ. Moreover, the current study indicates that alterations in sex hormones-mediated by MEQ may result from adrenal toxicity, adverse effects on the HPTA, and the dysfunction of Leydig cells, as well as the pathways responsible for steroid biosynthesis and cholesterol transport. Although the current study indicates that chronic exposure to MEQ has detrimental effects on TJs and spermatogenesis through alterations the functions of SCs and Leydig cells, and changing Y chromosome microdeletion gene expression, it still needs to be clearly inferred as to whether the fertility of exposed animals would be affected. Further investigation is absolutely required to clarify the adverse effects of chronic MEQ exposure on the fertility of male animals and to reveal the exact molecular mechanisms responsible for MEQ-induced changed levels of Y chromosome microdeletion genes and the dysfunction of SCs and Leydig cells.

## MATERIALS AND METHODS

### Chemical reagents

Mequindox (Molecular weight 218.21 g/mol, CAS No: 60875-16-3, purity 98 %) was obtained from Beijing Zhongnongfa Pharmaceutical Co. Ltd. (Huanggang, P.R. China). FSH, LH, GnRH and T kits were obtained from Nanjing Jiancheng Bioengineering Institute (Nanjing, P.R. China). All other chemicals were purchased from Sigma (St. Louis, USA) unless otherwise stated.

### Animals and diets

Forty male Kunming mice (6-7 weeks old, weighting 25-36 g) were obtained from Center of Laboratory Animals of Hubei Province (Wuhan, P.R. China). For each mouse, the individual body weight was within ± 20 % of the average. The room that was used to maintain mouse conditioned at 22 ± 3°C, a relative humidity of 50 % ± 20 %, and a 12-h light/dark cycle. The present study was conducted in accordance with the guidelines of the Committee on the Care and Use of Laboratory Animals of China (permit SYXK 20070044). All the mice were housed five per group in shoebox cages with hardwood shavings as bedding. Prior to dosing, signs of disease and weight gain were evaluated for each mouse by quarantined for 1 week.

After acclimatization, the male Kunming mice were randomly divided into four groups (*N* = 10). The control group administrated with the basic diet without additives. Three MEQ treated groups received the same diet supplemented with 25, 55 and 110 mg/kg MEQ, respectively. Food and water were supplied *ad libitum* and the treatment period lasted for 18 months. Symptoms and/or mortality were observed and carefully recorded each day during the 18-month period. All animal work was performed in compliance with the GLP-compliance and NIH publication “The Development of Science Based Guidelines for Laboratory Animal Care” [34].

### Experimental design

According to the Organization for Economic Cooperation and Development (OECD) Guideline 453 and Procedures for toxicological assessment of food in China, the high-dose level should cause some toxic effect performance or damage, and the low-dose group may not show any toxic effects, but should be 1-3 times greater than the clinical dose [64, 65]. Wang et al. reported that the LD_50_ values of MEQ in Kumming mice were 694.4 mg/kg b.w., using fixed dose procedure [66, 67]. In the present study, MEQ was incorporated in diets containing 0, 25, 55, 110 mg/kg, about equivalent to 0, 4, 8, 16 % of the LD_50_ in diets. In a previous sub-chronic and chronic toxicity study, MEQ in 110 mg/kg diet made an increase in plasma potassium (K^+^) level without growth inhibition [15]. Therefore, a dose of 110 mg/kg diet was selected as the high dose, and 55 mg/kg diet for the middle and 25 mg/kg diet for the low dose, respectively.

### Coefficients and preparation of testes

The mice were weighted and sacrificed. After weighing the body and testes, the coefficient of testes to body weight was calculated as the ratio of testes (wet weight, mg) to body weight (BW) (g). The testes were excised and quickly frozen at −70 °C after rinsing in phosphate buffered saline (PBS).

### Histopathological examination

The histopathological tests were performed using standard laboratory procedures. There was about half of testes fixed in Bouin's solution for 24 h. After fixation, the testes were rinsed by running water and embedded in paraffin blocks, then sliced into 5 μm sections, placed onto glass slides, and stained with hematoxylin-eosin (HE). Finally, morphological alterations in the testes were observed under an optical microscope (Olympus BX 41, Japan).

### Transmission electron microscope (TEM) observation

Testes were fixed by 2.5 % glutaraldehyde in 0.1 mol/dm^3^ cacodylate buffer for 4 h, followed by three washes with 0.1 mol/dm^3^ cacodylate buffer (pH 7.2-7.4), and then placed in 1 % osmium tetraoxide for 1 h. A graded series of ethanol (75, 85, 95, and 100 %) was used to dehydrate the specimens, which were then embedded in Epon 812. Ultra-thin sections (70 nm) were contrasted with lead citrate for 10 min and uranyl acetate for 30 min, and then observed with an H-7650 TEM (Hitachi, Japan).

### LC/MS–ITTOF analysis of MEQ and its metabolites in serum and testis

The prototype and metabolites of MEQ in the serum and testes were detected by hybrid IT/TOF mass spectrometry coupled to a high-performance liquid chromatography system (Shimadzu Corp., Kyoto, Japan). The liquid chromatography system (Shimadzu) was connected with a DGU-20A3 degasser, a photodiode array detector (SPD-M20A), a solvent delivery pump (LC-20AD), an autosampler (SIL-20AC), a communication base module (CBM-20A), and a column oven (CTO-20AC). The mass spectrometer was equipped with an electrospray ionization (ESI) source and operated in the positive mode.

#### Pretreatment methods of serum and testis

To detect the prototype and metabolites of MEQ in the serum, 200 μL of serum was mixed with 600 μL of extraction reagent (acetonitrile) and vortex-mixed for 5 min. Then, the solution was centrifuged at 10,000 × g for 15 min in an Omni mixer/homogenizer model 17106 (OMNI International, Waterbury, CT, USA) to collect the supernatant. The residual sediment was applied to extract MEQ and its metabolites following the above mentioned steps. The supernatant from the two extractions was merged and dried using N_2_ in a 40 °C water bath. After drying, the residue was dissolved with 200 μL solution of LC-MS/MS mobile phase [acetonitrile: 0.1 % formic acid (1:9, v/v)] to prepare the sample for LC/MS-IT-TOF analysis.

To detect the prototype and metabolites of MEQ in the testes, 1.0 g of the testis sample was ground and mixed with 5.0 mL of 40 °C 10 % trichloroacetic acid. After vigorous shaking, the homogenate was centrifuged at 10,000 × g for 15 min to collect the supernatant. A mixed reagent [dichloromethane: acetonitrile (2:1, v/v)] was used to extract MEQ and its metabolites twice. Then, 3.0 mL of mixed reagent was added to the supernatant and vortex-mixed for 5 min. After vigorous shaking, the solution was centrifuged at 10,000 × g for 15 min to obtain the lower liquid. Then, the lower liquid from the two extractions was merged and dried using N_2_ in a 40 °C water bath. The residue was reconstituted in 5 mL 5 % methanol. The reconstitution fluid was applied to the methanol (3.0 mL) and water (3.0 mL) pre-washed HLB 3cc cartridge (Waters Corporation, Milford, MA, USA). The reconstitution fluid was then sequentially washed with 3.0 mL of 5 % methanol in water and 5 mL of methanol. The extracts from the testis were eluted into plastic tubes using 5 mL of methanol and evaporated to dryness under N_2_ in a 40 °C water bath. After drying, the residue was dissolved in 500 μL of LC-MS/MS mobile phase [acetonitrile: 0.1 % formic acid (1:9, v/v)] and passed through a 0.22 μm filter membrane. The mixture (200 μL) was prepared for LC/MS-IT-TOF analysis.

#### Chromatographic and mass spectrometric conditions of MEQ and its metabolites

For chromatographic condition, the separation of MEQ and its metabolites in the serum and testis was performed on a VPODS column (150 × 2.0 mm; particle size, 5 μm) using a gradient elution consisting of mobile phase A (0.1 % formic acid in water) and mobile phase B (acetonitrile). The sample chamber in the autosampler was maintained at 4 °C, whereas the column was set at 40 °C. The gradient of the chromatographic condition was as follows: 0–5 min, linear gradient from 10 to 15 % B; 5–15 min, linear gradient to 70 % B; 15–18 min, linear gradient to 100 % B; 18–23 min, 100 % B; and 23–23.1 min, linear gradient back to 10 % B. The entire analysis was completed in 30 min. The 20 μL was applied as injection volume, and the flow rate was 0.2 mL/min.

For mass spectrometric condition, this analyses were carried out by full-scan MS with a mass range of 10–500 Da and data-dependent MS/MS acquisition on the suspected metabolites ions. Liquid nitrogen was used as the nebulizing gas at a flow rate of 1.5 L/min. The capillary and skimmer voltages were set at 4.5 kV and 1.6 kV, respectively. The CDL and heat block temperatures were both maintained at 200 °C. The MS^2^ spectra were produced using collision-induced dissociation (CID) of the selected precursor ions using argon as collision gas with relative energy of 50 %. The ion accumulation time were set at 50 ms, the precursor ion isolation width at 1 Da. The identification of MEQ and its metabolites were according to the recent researches [35, 1].

### Hormone assays

The serum sex hormone levels of FSH, LH, T and GnRH were measured by sandwich enzyme linked immunosorbent assay (ELISA) using specific commercial kits (Shanghai Dobio Biotech Co., LTD, Shanghai, P.R. China) according to the manufacturer's protocols.

### Quantitative real-time PCR

The mRNA expression of genes related to the synthesis of T (e.g. LH-R, 3β-HSD, 17β-HSD, CYP17, P450Scc, StAR and CYP19), spermatogenesis (e.g. INH-α, INH-β B, FSH-R), and Y chromosome (e.g. DDX3Y, HSF2, Ssty2 and Sly) in mouse testes were measured. The mRNA expression of these genes was determined by real-time quantitative reverse transcriptase-polymerase chain reaction (RT-PCR). Total RNA from the testes was extracted using Trizol reagent according to the manufacturer's instructions. One microgram of RNA was reverse transcribed to cDNA using the Rever^Tra^ Ace^TM^ First Strand cDNA Synthesis Kit (Promega, USA). Synthesized cDNA was used for quantitative real-time PCR (Bio-Rad, USA) with a SYBR^®^ Premix Ex Taq^TM^ RT-PCR kit (Takara, CodeDRR041 A, Japan).

Mouse specific primers were designed using Primer Express Software according to the software guidelines (Table 2). Each 25 μL reaction mixture consisted of 12.5 μL SYBR^®^ Premix Ex Taq^TM^, 1.0 μL of each primer (10 μm), 2.0 μL of cDNA, and 8.5 μL of RNase-free H_2_O. For 3β-HSD, 17β-HSD, CYP17, FSH-R, INH-α, INH-β B, LH-R, P450Scc, StAR, CYP19, DDX3Y, HSF2, Ssty2, and Sly, the cycling conditions were as follows: step 1, 30 s at 95°C; step 2, 45 cycles at 95°C for 5 s, 61 °C for 30 s; step 3, dissociation stage. In this study, the housekeeping gene β-actin was chosen as internal calibrator reference gene for the expression profiling of genes.

**Table 2:** PCR primers used in the gene expression analysis

Gene name	Description	Primer sequence (5′ - 3′)	Primer size (bp)
β-actin	mβ-actin-F	TTGCTGACAGGATGCAGAAG	141
	mβ-actin-R	ACATCTGCTGGAAGGTGGAC	
FSH-R	mFSHR-F	TTCTTGTGCCAATCCTTTCC	108
	mFSHR-R	TAAATCTGGGCTTGCACCTC	
LH-R	mLHR-F	ATGGCCATCCTCATCTTCAC	146
	mLHR-R	TTGGCACAAGAATTGACAGG	
INH-α	mINHα-F	GGTGGGGATCCTGGAATAAG	122
	mINHα-R	GCACCTGTAGCTGGGAAAAG	
INH-β B	mINHβB-F	TACGTGTGTCCAGAAGTGGC	111
	mINHβB-R	TTCGCCTAGTGTGGGTCAAC	
Ssty2	mSsty2-F	AGGTCAACTGCCAACAAACC	234
	mSsty2-R	ACCATCCCACTCCAGTTGTC	
DDX3Y	mDDX3Y-F	CCGGCCTGGACCAGCAATTTGT	239
	mDDX3Y-R	TGGGCTTCCCTCTGGAATCACGA	
Sly	mSly-F	ACGAACGAGAGAGGAAGAACA	210
	mSly-R	CCATGGACTTCTCATGCATTTC	
HSF2	mHSF2-F	AGGGGAGTACAACTGCATCG	106
	mHSF2-R	TTACTCTGGGTCGGTTCTGG	
StAR	mStAR-F	TGTCTCCCACTGCATAGCTG	103
	mStAR-R	TGTTCGTAGCTGCTGGTGTC	
P450scc	mP450scc-F	TTGGTTCCACTCCTCAAAGC	127
	mP450scc-R	CCAAAGTCTTGGCTGGAATC	
CYP17	mCYP17-F	GAGGATCCATAGCGCAGAAG	121
	mCYP17-R	GGGAGAGGAGAGGGAATTTG	
CYP19	mCYP19-F	CACATCATGCTGGACACCTC	98
	mCYP19-R	TGCCAGGCGTTAAAGTAACC	
3β-HSD	m3β-HSD-F	TGCAGACAAAGACCAAGGTG	106
	m3β-HSD-R	ACAGCAGCAGTGTGGATGAC	
17β-HSD	m17β-HSD-F	GATGTGGCTGTCAACTGTGC	101
	m17β-HSD-R	TTGATAACCCGCTGGAAGTC	

*Note*: The primers were manufactured by Nanjing Genescript Co. Ltd. (Nanjing, P.R. China). 17β-HSD, 17-beta-hydroxysteroid dehydrogenase; 3β-HSD, 3-beta-hydroxysteroid dehydrogenase; CYP17, cytochrome P450c17 subfamily a; CYP19, cytochrome P450c19 subfamily a; FSH-R, follicle-stimulating hormone receptor; INH-α, inhibin subfamily α; INH-β B, inhibin subfamily β B; LH-R, luteinizing hormone receptor; P450scc, cholesterol side-chain cleavage enzyme; StAR, steroidogenic acute regulatory protein.

Following amplification, the authenticity of the amplified product described as its specific melting temperature (Tm) was determined by a melt curve analysis with the complementary computer software. The threshold cycle of genes and the difference between their Ct values (ΔCt) were counted. The 2^−ΔΔCt^ data analysis method was used to calculate the relative quantitative levels of gene expression in accordance with the previous literature [1, 11, 14].

### Statistical analysis

All results are expressed as mean ± SD. Statistical analysis was performed using SPSS 15.0 software. Group differences were assessed by one-way analysis of variance followed by the least significance difference (LSD) test. *P*< 0.05 was considered statistically significant.

## Abbreviations

17β-HSD: 17-beta-hydroxysteroid dehydrogenase
3β-HSD: 3-beta-hydroxysteroid dehydrogenase
B-MEQ: bidesoxy-mequindox
BTB: blood-testis barrier
CBX: carbadox
CYP17: cytochrome P450c17 subfamily a
CYP19: cytochrome P450c19 subfamily a
FSH: follicle-stimulating hormone
FSH-R: follicle-stimulating hormone receptor
GnRH: gonadotropin-releasing hormone
HPTA: hypothalamic-pituitary-testicular axis
INH-α: inhibin subfamily α
INH-β B: inhibin subfamily β B
LC/MS-IT-TOF: high-performance liquid chromatography-mass spectrometry-ion trap-time-of-flight
LH: luteinizing hormone
LH-R: luteinizing hormone receptor
M4: 2-isoethanol 1-desoxymequindox
M8: 2-isoethanol 4-desoxymequindox
MEQ: mequindox
*N*1-MEQ: *N*1-desoxymequindox
OLA: olaquindox
P450scc: cholesterol side-chain cleavage enzyme
PBS: phosphate buffered saline
QdNOs: quinoxaline-di-*N*-oxides
SCs: Sertoli cells
StAR: steroidogenic acute regulatory protein
T: testosterone
TJs: tight junctions
